# Transfusion of Blood

**Published:** 1873-12

**Authors:** H. M. Madge


*OBSTETRICAL JOURNAL OF G. B. AND I.—November,* 1873:

ON TRANSFUSION OF BLOOD.—*H. M. Madge, M.D.*—Dr. Kingland made some remarks on transfusion, and related his experience. He believed the method by gravitation, as used by Dr. Macdonnell, to be the best.

The President had met with two cases of transfusion, both of which were fatal.

Dr. W. S. Playfair agreed with the author of the paper that the true value of the operation was not yet known. To gain this knowledge, it would be necessary to largely increase his number of cases in which the operation was practiced, and to carefully study the results. The apparatus must be simple and inexpensive, and ready at hand. As long as it was considered necessary to have a costly apparatus, the operation would never be popularized; and it would be postponed until, in nine cases out of ten, it would be too late. The method of transfusion with defibrinated blood was, he thought, the simplest, and the most likely to prove generally serviceable. The simplicity of Dr. Aveling's method was, he believed, more apparent than real, as it required some practice and delicate manipulation, and various circumstances would seriously militate against its success. No doubt Dr. Aveling could work his own contrivance easily, but he doubted if others could do it as well. The objections to the use of defibrinated blood were purely hypothetical. There seemed to him less fear of embolism than in other methods; and the operation could be done with the utmost calmness and deliberation. The experiments of Pancoast and Brown-Sequard had proved that the fibrine was quite useless in transfusion, the important element being the corpuscles. Borrowing Dr. Aveling's idea, he had had valves put to his instrument, which made it into a miniature Higginson's syringe; and so it could be used without any practice, and by the most uninstructed. Costing only a few shillings, it was within the reach of all, and might be carried in the corner of the obstetric bag ready for any sudden emergencies.

Dr. Barnes believed that, for the purpose of transfusion, the blood of animals was quite as efficient as human blood. The objection to the former might be said to be a sentimental one. No one would maintain that the blood of animals might not be taken into the human stomach, whilst the idea of swallowing human blood excited horror and disgust. The objections to Dr. Aveling's instrument had been overstated. All operations required dexterity and skill. It was certainly less bulky and less complicated than Dr. Kingland's apparatus. Valves were objectionable from their tendency to cause coagula, which might become detached and form small emboli. As to the relative merits of fibrinated and defibrinated blood, each had succeeded.

Fibrine was certainly not injurious, as its successful employment had shown. Time was an object; and defibrination involved loss of time. Transfusion was destined to a far greater extension, and would undoubtedly be employed in other diseases, such as puerperal convulsions, in which pure blood was required rather than an increased supply. The injection of saline fluids, which was a form of transfusion, had saved life. The form of apparatus, and the various questions as to the best mode of conducting the process, were questions which could only be determined by further experience.

Dr. Robert Macdonnell (Dublin) had operated in several cases, in three or four successfully. The results were much more favorable in patients of the well-to-do class. In the poorer class it scarcely ever succeeded. He approved of Dr. Aveling's instrument, but preferred his own, which comprised every possible requirement within a small compass. He regarded defibrination as an essential condition. Pure blood coagulated rapidly, and was liable to give rise to small emboli, which, he believed, were the invariable cause of those deaths which occurred a few days after transfusion. Fibrine was an excrementitious element, as shown by its absence in the blood which had passed through the great depurating organs of the body. Defibrinated blood contained all the elements necessary for temporary stimulus. It was physiologically better and surgically safer than pure blood. Defibrination need not necessarily cause delay.

Mr. Higginson (Liverpool) stated that his experience comprised fifteen cases of transfusion, of which ten were successful. His apparatus was not suitable for immediate transfusion. He always used pure blood. He highly approved of Dr. Aveling's instrument, and would certainly give it a trial. It might be used for intermediate as well as for immediate transfusion.

Dr. Kealy (Gosport) thought that, in the majority of cases, transfusion was out of the question for want of time.

Dr. Bird (Sydenham) wished to know which of the various forms of apparatus was the best.

Dr. Steele (Liverpool) said that Mr. Higginson's operations had shown the best results—namely, ten successful out of fifteen; and he (Mr. Higginson) expressed his opinion that Dr. Aveling's instrument would supersede all others.

Dr. Wiltshire (London) alluded to the cases of Dr. Martin, of Berlin, who had twelve successful cases, in all of which non-defibrinated blood was employed.

Dr. Aveling considered that transfusion was universally recognized as a legitimate operation; at present, however, none knew much about it, and we must therefore, speak with diffidence. The desiderata in apparatus were simplicity, small cost, and non-liability to get out of order. Syphilitic or other taints in the blood must be guarded against. The use of defibrinated blood involved delay, during which the patient might die.

